# Persistent critical illness in acute hypoxic respiratory failure: A retrospective cohort study

**DOI:** 10.1371/journal.pone.0358431

**Published:** 2026-09-16

**Authors:** Michael Kalinoski, Leah Bolden, Qi Wang, R. Adams Dudley, Nathan Mesfin, Nicholas E. Ingraham

**Affiliations:** 1 Department of Medicine, University of Minnesota Medical School, Minneapolis, Minnesota, United States of America; 2 Clinical and Translational Science Institute, University of Minnesota, Minneapolis, Minnesota, United States of America; 3 Center for Care Delivery and Outcomes Research (CCDOR) VA Minneapolis Healthcare System, Minneapolis, Minnesota, United States of America; Faculdade de Medicina de São José do Rio Preto, BRAZIL

## Abstract

**Introduction:**

Persistent critical illness (PerCI) describes patients with prolonged intensive care unit (ICU) dependence after their acute phase of illness and affects 5–20% of the general ICU population. PerCI has been studied in the general ICU cohort, but prevalence and outcomes remain unclear among mechanically ventilated patients with acute hypoxic respiratory failure (AHRF), a high-risk cohort that is more prone to ICU-associated complications. The objective of this study is to quantify PerCI prevalence, identify risk factors for PerCI development, and describe one-year mortality in this population.

**Methods:**

We conducted a retrospective study of patients with AHRF (PaO2/FiO2 ratio < 300) requiring mechanical ventilation within 72 hours of ICU admission across a 12-hospital health system (2011–2022). PerCI was defined as ICU length of stay ≥ 10 days. The primary outcome was one-year mortality, modeled using a Cox proportional hazards model with PerCI as a time-varying exposure. To identify risk factors for PerCI while accounting for competing early mortality, we used a dual-model approach: logistic regression comparing PerCI to early death (≤ 10-day mortality) and PerCI to early ICU discharge, with factors associated with both models considered robust predictors.

**Results:**

Among 10,626 eligible patients, 3,107 (29.2%) met criteria for PerCI. One-year survival was 64.2% in the PerCI group compared to 73.8% in the non-PerCI group. After PerCI onset, mortality risk increased threefold (hazard ratio 3.01; 95% CI 2.72–3.33). Factors consistently associated with PerCI across both models included ARDS, pneumonia, aspiration pneumonitis, heart failure, septic shock, weight loss, surgical admission, and postprocedural respiratory and circulatory failure.

**Conclusion:**

Nearly one in three mechanically ventilated AHRF patients developed PerCI, far exceeding rates in the general ICU populations and was associated with worse one-year survival. Identifiable clinical risk factors may support earlier goals-of-care conversations and post-ICU care planning in this high-risk population.

## Introduction

Persistent critical illness (PerCI) describes patients who survive the initial phase of critical illness but remain ICU-dependent because their recovery is delayed or fails entirely [1]. PerCI is thought to be the pathophysiological consequence of Persistent Inflammation, Immunosuppression, and Catabolism Syndrome (PICS), causing chronic inflammation and delayed clinical progression [2]. Although PerCI affects a minority of ICU patients (5–20%), it accounts for 40–60% of overall ICU costs [3–5]. Despite these high costs, their outcomes remain poor, with one-year mortality estimated to be between 40–67% [6]. Among those who survive to one year, fewer than 12% return to full independence [7,8]. Furthermore, given advances in ICU care delivery, PerCI rates are increasing [9]. PerCI’s associated high mortality, significant resource utilization, and increasing prevalence highlight the urgent need to proactively identify these high-risk subgroups to more effectively allocate post-ICU rehabilitation resources and improve their outcomes.

Most PerCI literature describes general ICU populations, with limited work in disease-specific cohorts such as sepsis (estimated PerCI prevalence of 2.8%) and frailty subgroups (4.3%) [1,10–12]. Acute hypoxic respiratory failure (AHRF) requiring invasive mechanical ventilation represents a clinically coherent population in which to study PerCI. These patients share a common pathophysiology of impaired gas exchange requiring ventilatory support, which creates compounding risks for prolonged ICU dependence. Mechanical ventilation itself causes iatrogenic complications (delirium, ventilator-associated events, hemodynamic instability), and hypoxemia can trigger multi-organ dysfunction that persists beyond the initial respiratory failure [13]. Prior work on prolonged mechanical ventilation does not fully capture this trajectory, because many patients remain ICU-dependent due to downstream complications despite liberation from the ventilator [12,14,15].

Despite these heightened risks, the prevalence of PerCI among mechanically ventilated AHRF patients, the clinical factors that predict its development, and its association with long-term survival remain uncharacterized. We aimed to quantify PerCI prevalence, identify risk factors for PerCI development, and describe one-year mortality in this population.

## Methods

### Study population & setting

We included adult patients (≥ 18 years of age) admitted to an ICU who required invasive mechanical ventilation within 72 hours of admission between January 2011 and December 2022. Our 12-hospital health system comprises both academic (n = 2) and community (n = 10) hospitals, with ICUs serving a mix of medical, surgical, cardiac, and mixed ICU patient populations. Only the initial ICU admission was included for patients with multiple ICU admissions during the study period. We excluded patients without a recorded PaO2 or with a P/F ratio >300 to enrich for intubations due to hypoxemia rather than airway protection. The P/F ratio was calculated by matching P_a_O_2_ values with the closest F_i_O_2_ recorded within a ± 1-hour window. For patients with multiple P/F ratios, we used the lowest daily P/F ratio. This study was approved by the University of Minnesota Institutional Review Board (STUDY00014815).

### Data collection

We abstracted the following from electronic health records: age, sex, self-reported race/ethnicity (Asian, Black or African American, non-Hispanic White, All Others), admission code status, primary service (medicine vs. surgical), ICU source (emergency department vs. general ward), admission diagnoses (identified using ICD-10 codes; S1 Table), Elixhauser comorbidities [16], Laboratory Acute Physiology Score version 2 (LAPS2, admission worst) [17], tobacco use, worst daily P/F ratio, duration of invasive mechanical ventilation, and discharge disposition. Mortality data is provided by the Department of Health on a monthly basis and ensures we capture deaths that occur after hospitalization, in addition to those that occur during.

### Statistical analysis

Descriptive statistics were calculated and presented as the mean and standard deviation (SD) for normally distributed continuous variables, the median and interquartile range (IQR) for non-normally distributed continuous variables, and frequencies and percentages for categorical variables. Groups were compared using analysis of variance (ANOVA) for continuous variables that are normally distributed, Kruskal-Wallis test for continuous variables that are not normally distributed, and Chi-square tests for categorical variables. Kaplan-Meier survival curves were constructed and a log-rank test was used to compare unadjusted survival between patients with PerCI and those without. Missing data were minimal, less than 5% for all covariates, and were handled using complete-case analysis (S2 Table). Statistical analyses were performed in SAS 9.4 (SAS Institute Inc., Cary, NC). P values of less than 0.05 were considered statistically significant.

### PerCI definition

PerCI conceptually represents a state where patients have survived the acute phase of their illness but remain ICU-dependent due to complications that develop during their stay, rather than their original presenting illness. We defined PerCI as an ICU length of stay >=10 days, consistent with prior literature. This threshold has been empirically validated across multiple cohorts as the point which the predictive value of acute illness characteristics progressively declines until it becomes lower than that of antecedent patient characteristics [1,10,12,18]. We acknowledge that some patients with AHRF, particularly those with severe ARDS, may not have resolved their initial acute illness by day 10. However, our prior work validated this definition in our general ICU population from the same health system [18]. Moreover, this concern motivated the dual-model approach described below, which distinguishes factors associated with prolonged ICU dependence from those reflecting ongoing acute severity. While we chose 10 days for our primary definition, other studies have found that the PerCI definition for the general ICU population, and some subgroups, may be better defined at a shorter timepoint [10,11]. To test if our findings were limited to our 10-day definition, we performed a sensitivity analysis using a 7-day cutoff. The rational for use of a 7-day threshold for our sensitivity analysis was this cutoff was identified as a transition point in the sensitivity analysis of a previous study focusing on the PerCI respiratory failure population [10].

### Model 1: association between PerCI and one-year mortality

A Cox proportional hazards model was used to measure factors associated with 1-year mortality. Candidate covariates were prespecified based on prior literature and selected using stepwise methods to limit overfitting. Our initial set of covariates included age, gender, race, tobacco use, ICU source, primary service, index initial code status, hospital length of stay, pre-ICU length of stay, Elixhauser score, LAPS2 Score admission worst, PerCI, ventilator days, discharge disposition, and hospice discharge. The proportional hazards assumption was assessed using Schoenfeld residuals. Our main exposure was PerCI, defined as an ICU length of stay of 10 days or longer. The primary outcome was one-year mortality, ascertained through linkage with the Department of Health vital records for deaths that occur outside of our facility. Mortality was measured from the date of ICU admission to allow standardized comparisons. We included PerCI as a time-varying exposure in the model to account for immortal time bias [19]. By modeling PerCI as a time-varying exposure, patients contribute person-days to the PerCI group only after meeting the PerCI criteria (i.e., ICU stay ≥10 days), thereby preventing the misattribution of survival time before PerCI onset to the exposed group and reducing immortal time bias. To prevent the introduction of overadjustment or collider bias, post-PerCI variables (ICU LOS, vent days, discharge disposition) were excluded a priori as potential mediators on the causal pathway between PerCI and mortality as demonstrated in S1 Fig.

### Model 2: risk factors for PerCI development

Identifying risk factors for PerCI is complicated by the fact that patients who die before day 10 cannot develop PerCI, creating a competing risk. To address this, we used two complementary logistic regression models.

- Model 2A (PerCI vs. Early Death): Among patients who either developed PerCI or died within 10 days (excluding those discharged alive before day 10), this model identified factors associated with surviving to day 10 with ongoing ICU dependence rather than early mortality.
- Model 2B (PerCI vs. Early Discharge): Among patients who either developed PerCI or were discharged from the ICU within 10 days (excluding those who died before day 10), this model identified factors associated with prolonged ICU dependence among survivors.

Risk factors significantly associated with PerCI in both models, in the same direction, were considered robust predictors of survival beyond the acute phase and prolonged ICU dependency. This dual-model approach captures the entire cohort while identifying factors specifically associated with the PerCI phenotype rather than merely acute illness severity. We avoid directly comparing patients who couldn’t have developed PerCI (because they died early) to those who survived and did not develop PerCI, while also still accounting for all the patients across both models, thereby mitigating immortal time bias. This two-comparison approach distinguishes factors associated with surviving to day 10 with ongoing ICU dependence from factors associated with prolonged ICU dependence among survivors [20]. We chose complementary logistic models over competing risk regression (e.g., Fine-Gray) because the resulting odds ratios are more clinically interpretable and the two-model framework maps directly onto clinicians’ sequential reasoning: first, will this patient survive the acute phase? Second, if they survive, will they recover or remain ICU-dependent? Factors that were statistically significant in both models suggest a phenotype that survives but recovers slowly, consistent with the conceptual definition of PerCI.

## Results

### Patient characteristics

**Fig 1:**
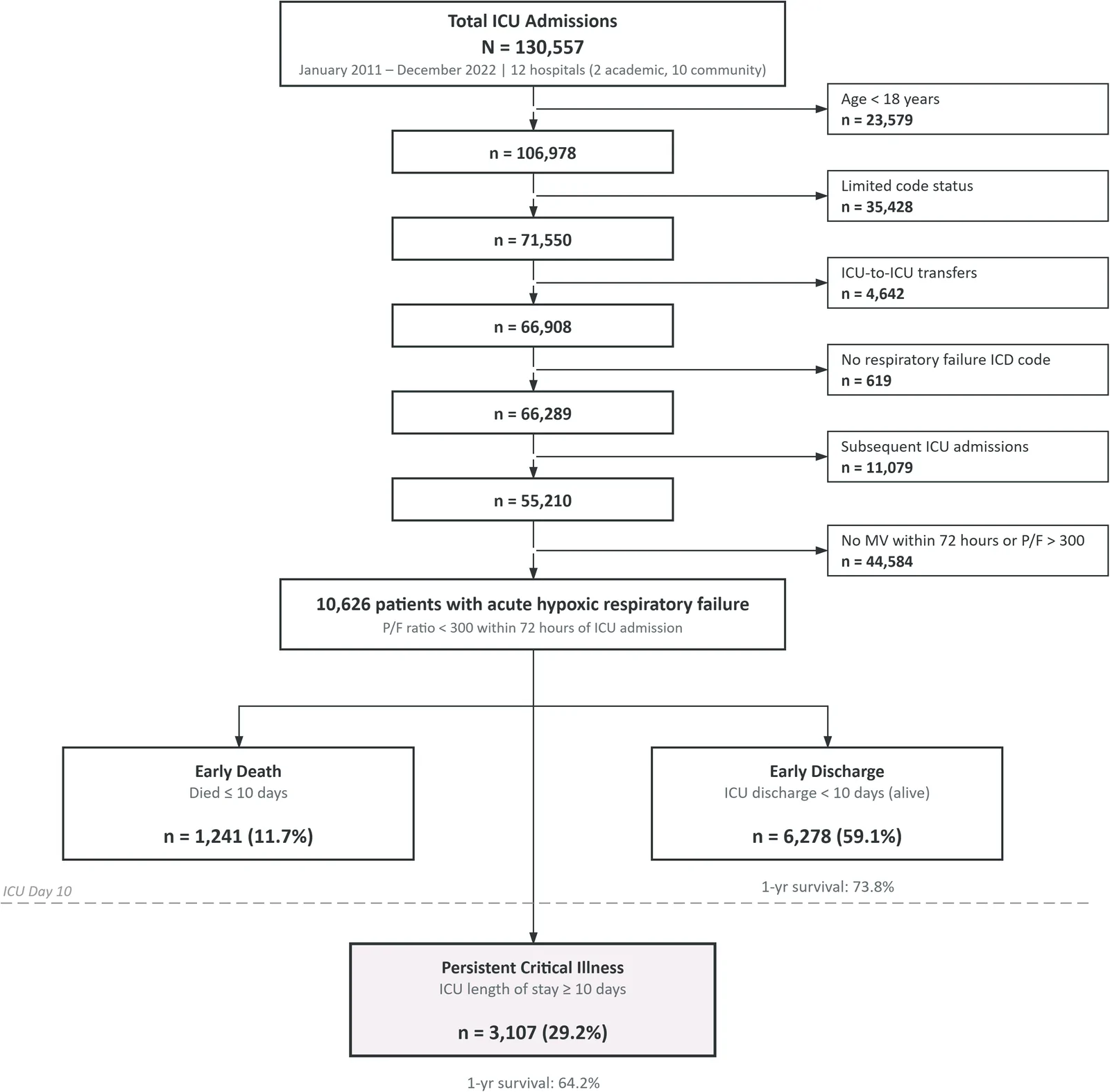
Final study population after applying inclusion and exclusion criteria.

Among 10,626 eligible patients, 3,107 (29.2%) met criteria for PerCI (Fig 1). The mean age was 60.9 years (SD 15.9), 58.0% were male, and 81.5% were non-Hispanic White. The majority were admitted from the emergency department (59.1%) and under surgical services (54.8%). The median duration of mechanical ventilation was 2 days (IQR 1–7), reflecting a large proportion of patients with brief intubations; among PerCI patients, the median was 10 days (IQR 6–18). Patients who developed PerCI had higher illness severity at presentation than those discharged before day 10 (mean LAPS2 160.2 vs 145.0), though patients who died early had the highest severity (LAPS2 203.5; p < .0001 across groups). PerCI patients had greater comorbidity burden (median Elixhauser score 8 vs 6, p < .0001) and sepsis was more prevalent in both the PerCI (59.8%) and early death (64.3%) groups compared to the non-PerCI patients (29.3%; p < .0001). Among PerCI patients, in-hospital mortality was 24.9% and one-year mortality was 35.8%, compared to 2.4% and 11.7% in non-PerCI patients (p < .0001). Full baseline characteristics are presented in Table 1 (complete data in S3 Table).

**Table 1:** Baseline characteristics of patients with acute hypoxic respiratory failure stratified by PerCI status (early death, PerCI, no PerCI).

Variable	All Patients(N = 10,626)	Early Death(N = 1,241)	PerCI(N = 3,107)	No PerCI(N = 6,278)	P Value
**Demographics**					
Age, mean (SD)	60.9 (15.9)	66.6 (15.4)	59.6 (15.3)	60.4 (16.0)	<.0001
Female, n (%)	4,467 (42.0)	581 (46.8)	1,302 (41.9)	2,584 (41.2)	0.001
Race, n (%)					0.039
Asian	553 (5.2)	61 (4.9)	186 (6.0)	306 (4.9)	
Black or African American	783 (7.4)	84 (6.8)	255 (8.2)	444 (7.1)	
Other/Unknown	627 (5.9)	84 (6.8)	186 (6.0)	357 (5.7)	
Non-Hispanic White	8,663 (81.5)	1,012 (81.5)	2,480 (79.8)	5,171 (82.4)	
**Comorbidities**					
Elixhauser Score, median (IQR)	7 (0–16)	10 (0–20)	8 (0–16)	6 (0–14)	<.0001
Congestive Heart Failure	2,058 (19.4)	243 (19.6)	657 (21.1)	1,158 (18.4)	0.008
Neurological Disorder	2,680 (25.2)	323 (26.0)	844 (27.2)	1,513 (24.1)	0.005
Renal Failure	1,953 (18.4)	313 (25.2)	592 (19.1)	1,048 (16.7)	<.0001
Liver Disease	1,526 (14.4)	234 (18.9)	460 (14.8)	832 (13.3)	<.0001
Metastatic Cancer	841 (7.9)	155 (12.5)	200 (6.4)	486 (7.7)	<.0001
Weight Loss	1,389 (13.1)	188 (15.1)	518 (16.7)	683 (10.9)	<.0001
Fluid & Electrolyte Disorder	3,745 (35.2)	541 (43.6)	1,211 (39.0)	1,993 (31.8)	<.0001
**Admission Diagnoses, n (%)**					
Cardiac Arrest	1,206 (11.3)	411 (33.1)	379 (12.2)	416 (6.6)	<.0001
Heart Failure	3,166 (29.8)	385 (31.0)	1,077 (34.7)	1,704 (27.1)	<.0001
Post-Proc Resp/Circ Failure	920 (8.7)	36 (2.9)	458 (14.7)	426 (6.8)	<.0001
COVID-19	533 (5.0)	73 (5.9)	331 (10.7)	129 (2.1)	<.0001
Respiratory Failure	7,483 (70.4)	1,017 (82.0)	2,529 (81.4)	3,937 (62.7)	<.0001
ARDS	515 (4.8)	67 (5.4)	354 (11.4)	94 (1.5)	<.0001
Pneumonia	3,666 (34.5)	410 (33.0)	1,758 (56.6)	1,498 (23.9)	<.0001
COPD	1,895 (17.8)	232 (18.7)	570 (18.3)	1,093 (17.4)	0.377
Asthma	908 (8.5)	60 (4.8)	218 (7.0)	630 (10.0)	<.0001
Aspiration Pneumonitis	1,618 (15.2)	222 (17.9)	623 (20.1)	773 (12.3)	<.0001
Septic Shock‡	4,498 (42.3)	798 (64.3)	1,858 (59.8)	1,842 (29.3)	<.0001
**Clinical Variables & Outcomes**					
ICU Source: Ward, n (%)	4,341 (40.9)	480 (38.7)	1,359 (43.7)	2,502 (39.9)	0.0004
Primary Service: Surgery, n (%)	5,828 (54.8)	398 (32.1)	1,680 (54.1)	3,750 (59.7)	<.0001
LAPS2, mean (SD)	156.3 (52.0)	203.5 (52.9)	160.2 (52.6)	145.0 (45.7)	<.0001
Full Code, n (%)	8,774 (82.6)	439 (35.4)	2,400 (77.2)	5,935 (94.5)	<.0001
MV Duration, median days (IQR)	2 (1–7)	3 (1–5)	10 (6–18)	1 (1–3)	<.0001
Hospital LOS, median days (IQR)	11 (6–19)	5 (2–8)	22 (16–31)	9 (6–14)	<.0001
In-Hospital Mortality, n (%)	2,147 (20.2)	1,224 (98.6)	773 (24.9)	150 (2.4)	<.0001
One-Year Mortality, n (%)	3,086 (290)	1,241 (100.0)	1,113 (35.8)	732 (11.7)	<.0001

Footnotes: For categorical variables, we used chi-square test. For continuous variables, we used ANOVA if normally distributed and Kruskal-Wallis test if not normally distributed. Elixhauser comorbidities shown are those significant across groups or retained in multivariable models; full comorbidity associations available in S1 Table. ‡ Septic Shock = the “Sepsis Plus” category of the three-level variable (No Sepsis/Shock; Sepsis Only, n = 252, 2.4%; Septic Shock). Abbreviations: ARDS – Acute Respiratory Distress Syndrome; Circ – Circulatory; IQR – Interquartile Range; LAPS2 – Laboratory-based Acute Physiology Score, Version 2; LOS – Length of Stay; MV – Mechanical Ventilation; SD – Standard Deviation.

### One-year mortality (model 1)

**Fig 2:**
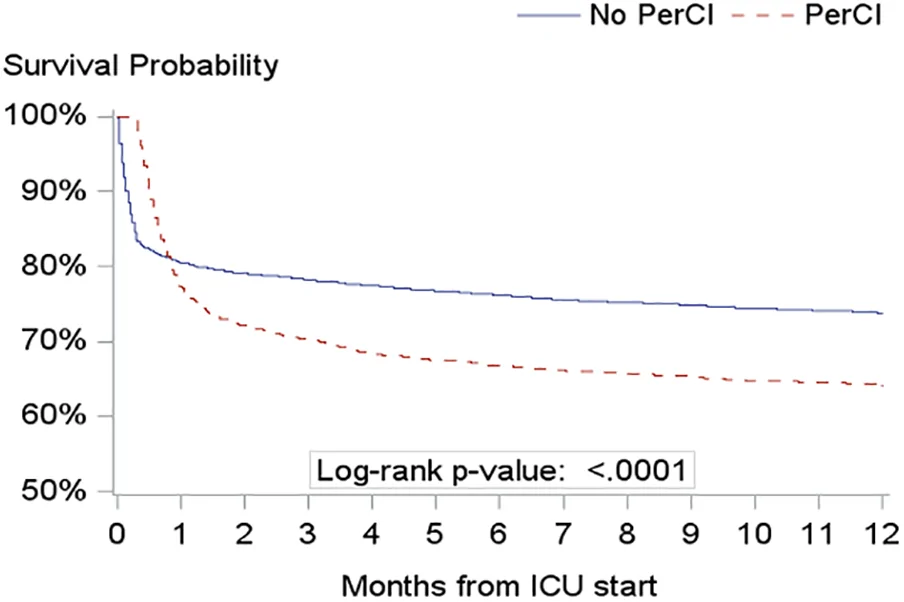
Kaplan-meier survival curves comparing PerCI and non-PerCI cohorts over one year.

One-year survival was 64.2% in the PerCI group compared to 73.8% in the non-PerCI group (Fig 2). In the adjusted Cox model with PerCI as a time-varying exposure, PerCI was associated with a threefold increase in mortality risk (HR 3.01; 95% CI 2.72–3.33). Because the time-varying approach attributes risk only after PerCI onset (day 10), the HR reflects the attributable mortality risk solely related to PerCI status. Other factors independently associated with one-year mortality included age (HR 1.03 per year; 95% CI 1.03–1.03), cardiac arrest (HR 2.22; 95% CI 2.01–2.45), medicine service (HR 1.98; 95% CI 1.81–2.16), and COVID-19 (HR 1.40; 95% CI 1.20–1.63). Full model results are in Table 2 (including hospital-level results in S4 Table).

**Table 2:** Cox proportional hazards model: factors associated with one-year mortality among mechanically ventilated patients with acute hypoxic respiratory failure.

Parameter	Level	HR (95% CI)	P Value
Age (per year)		1.03 (1.03–1.03)	<.0001
***Race (ref: White)***			
	Asian	1.06 (0.91–1.25)	0.453
	Black	0.95 (0.82–1.10)	0.470
	Other/Unknown	1.22 (1.05–1.42)	0.010
***Tobacco Use (ref: Never)***			
	Quit	1.06 (0.97–1.16)	0.229
	Unknown	0.85 (0.75–0.95)	0.006
	Yes	1.06 (0.93–1.20)	0.377
***Elixhauser Comorbidities***			
Hypertension		0.87 (0.80–0.96)	0.003
Neurological Disorder		0.90 (0.82–0.98)	0.013
Diabetes W/O Chronic Comp		0.87 (0.78–0.98)	0.025
Diabetes W/ Chronic Comp		0.83 (0.75–0.92)	<.001
Renal Failure		1.20 (1.09–1.32)	0.0002
Liver Disease		1.15 (1.03–1.28)	0.011
Lymphoma		1.35 (1.12–1.63)	0.002
Metastatic Cancer		1.46 (1.31–1.64)	<.0001
Coagulation Deficiency		1.13 (1.02–1.26)	0.018
Fluid & Electrolyte Disorder		1.12 (1.03–1.22)	0.012
Deficiency Anemia		1.11 (1.02–1.22)	0.021
Alcohol Abuse		0.88 (0.79–0.99)	0.040
***Admission Dx Category (ref: Cardiovascular)***			
	Endocrine/Metabolic/Immunity	0.77 (0.61–0.96)	0.021
	Factors Influencing Health	0.49 (0.42–0.58)	<.0001
	GI	1.11 (0.94–1.29)	0.212
	ID	1.46 (1.28–1.68)	<.0001
	Injury/Poisoning	1.10 (0.90–1.36)	0.352
	Neurology	0.76 (0.61–0.93)	0.008
	Oncology	1.45 (1.21–1.73)	<.0001
	Other	1.01 (0.89–1.16)	0.835
	Pulmonary	0.91 (0.80–1.04)	0.146
	Signs/Symptoms/Abn Findings	0.83 (0.72–0.96)	0.014
***Specific Diagnoses***			
Cardiac Arrest		2.22 (2.01–2.45)	<.0001
Post-Proc Resp/Circ Failure		0.73 (0.64–0.84)	<.0001
COVID-19		1.40 (1.20–1.63)	<.0001
Respiratory Failure		1.19 (1.07–1.32)	0.001
ARDS		1.32 (1.13–1.55)	<.001
Pneumonia		0.92 (0.85–1.00)	0.049
COPD		1.12 (1.02–1.23)	0.023
Asthma		0.65 (0.54–0.77)	<.0001
***Other Covariates***			
ICU Source	Ward vs ED	1.15 (1.06–1.25)	0.001
Primary Service	Medicine vs Surgery	1.98 (1.81–2.16)	<.0001
LAPS2 (per point)		1.008 (1.007–1.008)	<.0001
Pre-ICU LOS (per day)		1.03 (1.02–1.03)	<.0001
PerCI (time-varying)	Yes vs No	3.01 (2.72–3.33)	<.0001

PerCI modeled as a time-varying exposure. vent_days and elix_score (composite) were not included per statistical analysis plan. Hospital-level results available in Supplemental Table 2. Abbreviations: Abn – Abnormal; CI – Confidence Interval; Dx – Diagnosis; ED – Emergency Department; GI – Gastrointestinal; HR – Hazard Ratio; ID – Infectious Disease; LAPS2 – Laboratory-based Acute Physiology Score, Version 2; LOS – Length of Stay; Proc – Procedural; Resp – Respiratory; Circ – Circulatory.

### Risk factors for PerCI (model 2)

**Fig 3:**
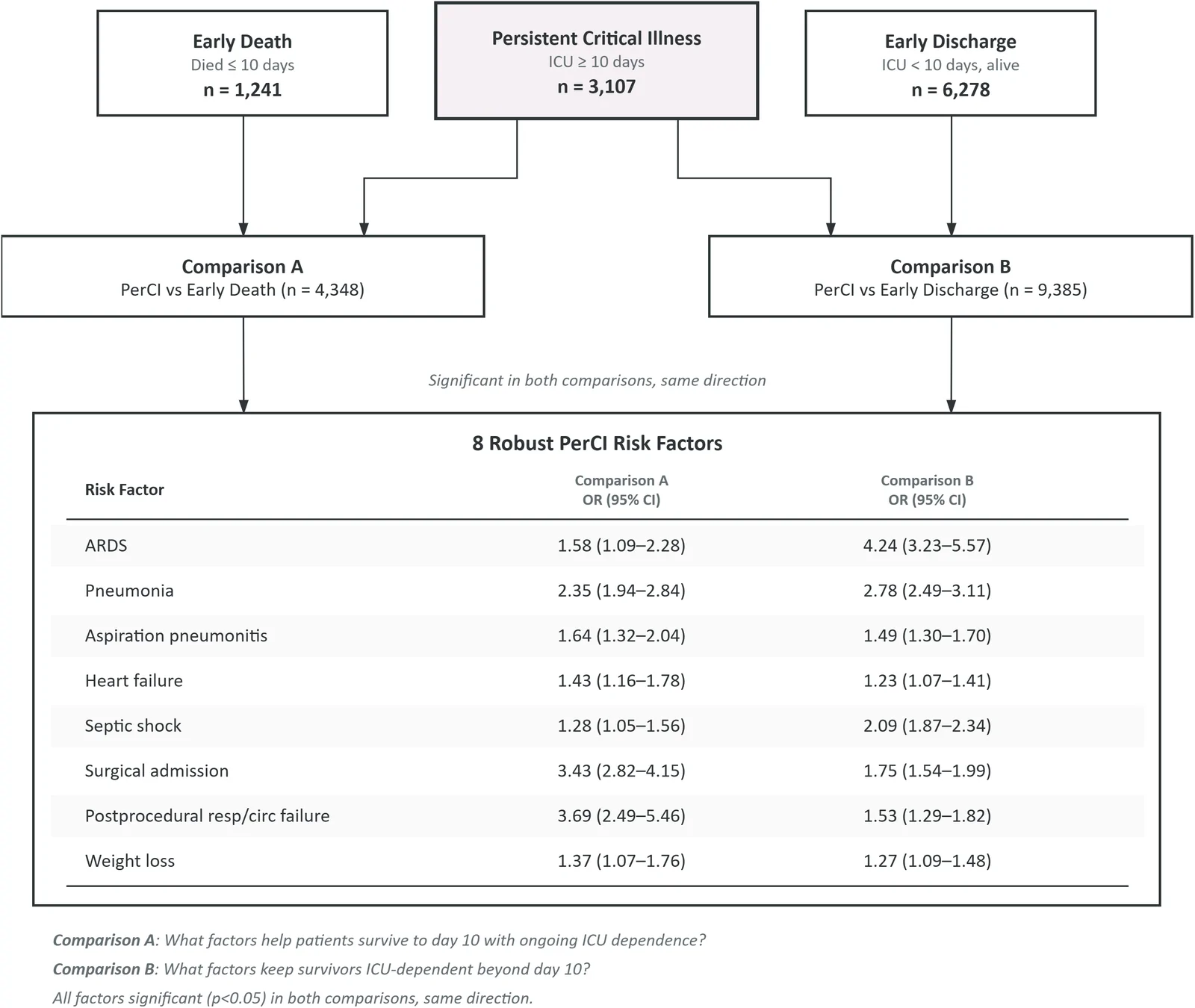
Dual-model approach for identifying robust PCI risk factors.

Eight clinical factors were independently associated with PerCI in both Model 2A and 2B (S5 Table; Fig 3). ARDS had the strongest association with PerCI versus no PerCI among survivors (OR 4.24; 95% CI 3.23–5.57) and was also significant versus early death (OR 1.58; 95% CI 1.09–2.28). Postprocedural respiratory/circulatory failure had the strongest association with PerCI versus early death (OR 3.69; 95% CI 2.49–5.46), followed by surgical admission (OR 3.43; 95% CI 2.82–4.15). The remaining five factors (pneumonia, aspiration pneumonitis, heart failure, septic shock, and weight loss) were significant in both comparisons (Fig 3). These eight overlapping risk factors were considered to be robust predictors of PerCI given our approach to mitigate immortal time bias.

The sensitivity analysis using a 7-day cutoff was completed as a robustness check. This demonstrated a similar mortality association (time-varying HR 2.67; 95% CI 2.40–2.96 vs 3.01; 95% CI 2.72–3.33, S6 Table) despite nearly doubling PerCI prevalence (44.3% vs 29.2%, S7 Table).

## Discussion

Nearly one in three mechanically ventilated patients with AHRF developed PerCI in our cohort, which is 5–10 times higher than reported in general ICU populations using the same 10-day threshold [1,10,12]. Patients with PerCI had lower one-year survival than those without PerCI (64.2% vs. 73.8%), and significant risk factors for PerCI included: ARDS, aspiration pneumonitis, heart failure, pneumonia, septic shock, weight loss, surgical admission, and postprocedural respiratory and circulatory failure.

### PerCI prevalence

PerCI is conceptually distinct from chronic critical illness (CCI), which is traditionally defined by prolonged mechanical ventilation alone [4,7]. Iwashyna et al. proposed that PerCI begins when baseline patient characteristics surpass acute illness severity in predicting outcomes, a transition that regression modeling has consistently identified at approximately 10 days across multiple cohorts [1,10,12,21]. This distinction matters because it broadens the definition from patients on prolonged mechanical ventilation to a larger ICU population that remains ICU-dependent beyond their acute admitting illness. Importantly, in our sensitivity analysis, we found that decreasing the definition to 7 days almost doubles the prevalence of PerCI. While our results in this study were robust to the definition, future studies will be required to best operationalize a standard definition that appropriately captures the complex PerCI population.

The substantially higher PerCI prevalence in AHRF may reflect two mechanisms. First, mechanical ventilation itself often generates complications that delay recovery (ventilator-associated events, sedation-related delirium, and hemodynamic instability), creating a self-reinforcing cycle of ICU dependence [22]. Second, hypoxemia can trigger multi-organ dysfunction that persists beyond the initial respiratory failure; patients with ARDS, for example, often die from multi-organ failure with sepsis as the triggering event than from refractory hypoxemia [23]. We hypothesize both mechanisms prolong ICU stays beyond the original indication for admission.

This prevalence is notably higher than PerCI in sepsis (2.8%) or frailty (4.3%) subgroups [11,12], likely because our cohort (mechanically ventilated patients with confirmed hypoxemia) is enriched for higher severity of illness on presentation. In contrast, general ICU populations include many patients admitted for monitoring or brief interventions who are discharged within days. The value of studying PerCI in AHRF is therefore not the prevalence estimate itself, but the identification of which patients within this high-risk group develop PerCI and its implication on clinical decision-making, such as early rehabilitation [24].

### Mortality in PerCI

Despite the higher PerCI prevalence in AHRF, one-year survival among PerCI patients (64.2%) fell within the range reported for general ICU populations (40–67%) [3]. This convergence suggests that the clinical trajectory of PerCI may be driven more by complications that accumulate during the ICU stay than by the index condition, consistent with the conceptual framework that PerCI represents a distinct clinical state where baseline characteristics supersede acute illness in predicting outcomes [21]. Mortality alone underestimates PerCI’s impact: prior work suggests fewer than 12% of PerCI survivors return to full independence [7,25].

### PerCI risk factors

The eight factors associated with PerCI appear to cluster into three mechanistic categories: conditions that prolong ventilatory dependence (ARDS, pneumonia, aspiration pneumonitis), conditions reflecting baseline vulnerability and multi-organ complications (heart failure, septic shock, weight loss), and markers of surgical or procedural complexity with concurrent organ dysfunction (surgical admission, postprocedural respiratory/circulatory failure). We hypothesize that the dual-model requirement means these factors are not merely markers of severity; they are associated with both surviving the acute phase but failing to recover quickly from it.

The strong association between ARDS and PerCI versus early discharge (OR 4.24) is consistent with its known course: ARDS patients require prolonged mechanical ventilation, as demonstrated across landmark trials [26–28], directly overlapping with the PerCI threshold. The remaining respiratory factors (aspiration pneumonitis, pneumonia) may reflect impaired airway protection and multi-organ dysfunction that prolong ICU dependence without necessarily increasing the acute risk of death [29,32]. Heart failure represents a condition in which an acutely reversible process, such as pulmonary edema requiring mechanical ventilation, may occur in the setting of an underlying chronic debility or cardiogenic shock state that likely prolongs ICU dependence [30,31]. Septic shock is a heterogeneous syndrome in which some patients experience a transient shock state, while others suffer a prolonged course that increases the risk of ICU dependence [32]. The association with surgical admission type likely reflects the acute, corrective nature conferred by surgical procedures. Additionally, it highlights the high-risk profile and invasiveness of the subset of surgeries that necessitate continued mechanical ventilation postoperatively. Weight loss as a predictor aligns with evidence that cachexia can impair respiratory muscle function, ventilator liberation and overall rehabilitation, slowing recovery without directly contributing to mortality [33].

Our dual-model approach provides a clinically intuitive framework for assessing PerCI-specific predictors that acknowledges both the possibility of early death and early discharge, events that are mutually exclusive to PerCI. A factor associated with PerCI in only one comparison might reflect severity (predicting survival but not prolonged dependence) or frailty (predicting prolonged dependence but not survival). Factors significant in both comparisons identify the specific phenotype that survives the acute phase but fails to recover.

PerCI creates tension between providers and families about expected outcomes, compounded by evidence that chronic critical illness is poorly understood by patients’ surrogates [34,35]. Our findings provide a framework for earlier, more specific prognostic conversations. Clinicians can assess early during hospitalization whether a patient carries multiple PerCI risk factors and proactively frame expectations. For patients or surrogates who have expressed strong preferences against prolonged ICU dependence or loss of independence, this risk stratification can anchor shared decision-making at a time when goals-of-care conversations are most productive (i.e., early), before the trajectory is established.

Beyond prognostication, early identification of PerCI risk could trigger proactive rehabilitation planning, including early mobility protocols, nutrition optimization, and identification of post-acute care needs before they become self-evident. Further research is needed to identify specific interventions associated with improved functional outcomes after ICU dependence.

### Generalizability & limitations

This study was conducted within a single health system, which may limit generalizability; however, the inclusion of both academic and community-based hospitals enhances the overall representativeness of our findings. Our cohort was older and predominantly non-Hispanic white, which may limit generalizability to more diverse populations [36]. Residual confounding may account for systematic unaccounted differences between PerCI and non-PerCI patients; however, we used a priori-specified variables derived from granular EHR data to minimize this limitation, which remains an inherent challenge in all retrospective observational studies

## Conclusions

PerCI affected 30% of mechanically ventilated AHRF patients, exceeding rates in general ICU populations. PerCI was associated with a threefold increase in post-onset mortality risk and eight identifiable clinical factors were associated with PerCI across two complementary analytic models. These findings can inform earlier prognostic conversations and post-ICU care planning. Prospective studies are needed to determine whether early PerCI risk stratification improves patient-centered outcomes, including functional independence, readmission, and post-intensive care syndrome.

## Supporting information

S1 Table ICD-10 Diagnosis Codes Used for Admission Diagnosis Classification. Diagnosis categories based on primary ICD-10 admission diagnosis codes. Code ranges shown are simplified; see full code lists in study protocol.
(DOCX)

S1 Fig Directed Acyclic Graph (DAG) for the primary Cox Model. (TIFF)

S2 Table Missing Data. (DOCX)

S3 Table Complete Baseline Characteristics of Patients with Acute Hypoxic Respiratory Failure Stratified by PCI Status (Early Death, PCI, No PCI). (DOCX)

S4 Table Complete Cox Proportional Hazards Model: Factors Associated with One-Year Mortality Among Mechanically Ventilated Patients with Acute Hypoxic Respiratory Failure, Including Hospital-Level Results. PCI modeled as a time-varying exposure. vent_days and elix_score (composite) were not included per statistical analysis plan. This table includes hospital-level results omitted from Table 2 for brevity. Abbreviations: Abn – Abnormal; CI – Confidence Interval; Dx – Diagnosis; ED – Emergency Department; GI – Gastrointestinal; HR – Hazard Ratio; ID – Infectious Disease; LAPS2 – Laboratory-based Acute Physiology Score, Version 2; LOS – Length of Stay; Proc – Procedural; Resp – Respiratory; Circ – Circulatory; UMMC – University of Minnesota Medical Center.
(DOCX)

S5 Table Multivariable Logistic Regression: Risk Factors for PCI Development. Model 2A compares PCI to Early Death (≤10 days); Model 2B compares PCI to Early ICU Discharge (≤10 days). Model 2A: PCI vs. Early Death (≤10-day mortality). Model 2B: PCI vs. Early ICU Discharge (≤10-day survivors). vent_days and elix_score (composite) were not included. Hospital-level results omitted. Abbreviations: Abn – Abnormal; CHF – Congestive Heart Failure; CI – Confidence Interval; CV – Cardiovascular; Dx – Diagnosis; ED – Emergency Department; GI – Gastrointestinal; ID – Infectious Disease; Infl – Influencing; LAPS2 – Laboratory-based Acute Physiology Score, Version 2; LOS – Length of Stay; OR – Odds Ratio; PAD – Peripheral Artery Disease; Proc – Procedural; Pulm – Pulmonary; Resp – Respiratory; Circ – Circulatory; Sx – Symptoms.
(DOCX)

S6 Table Sensitivity Analysis of Cox Proportional Hazards Model Using a 7 day Cutoff for PerCI. (DOCX)

S7 Table Sensitivity Analysis of Final Study Population Using a 7-day & 10-day Cutoff for PerCI. (DOCX)
