# Supplementary material for: Persistent critical illness in acute hypoxic respiratory failure: A retrospective cohort study
Source: PLoS One. 2026 Sep 16;21(9):e0358431. doi: 10.1371/journal.pone.0358431 (PMC13580957; doi:10.1371/journal.pone.0358431)
Supplement: Supplementary file 1 [file pone.0358431.s001.docx]

| **Diagnosis Category** | **ICD-10 Code Range** |
| --- | --- |
| Infectious Disease | A00–B99, U07 |
| Oncology | C00–D49 |
| Hematology | D50–D89 |
| Endocrinology | E00–E89 |
| Psychiatry | F01–F99 |
| Neurology | G00–G99 |
| Eye | H00–H59 |
| Ear | H60–H95 |
| Cardiovascular | I00–I99 |
| Pulmonology | J00–J99 |
| Gastrointestinal | K00–K95 |
| Dermatology | L00–L99 |
| Musculoskeletal | M00–M99 |
| Genitourinary | N00–N99 |
| Gynecological | O00–O9A |
| Perinatal | P00–P96 |
| Congenital | Q01–Q99 |
| Signs/Symptoms/Abnormal Findings | R00–R99 |
| Injury/Poisoning | S00–T88 |
| External Causes of Mortality | V00–Y99 |
| Factors Influencing Health Status | Z00–Z99 |
