# Supplementary material for: Persistent critical illness in acute hypoxic respiratory failure: A retrospective cohort study
Source: PLoS One. 2026 Sep 16;21(9):e0358431. doi: 10.1371/journal.pone.0358431 (PMC13580957; doi:10.1371/journal.pone.0358431)
Supplement: Supplementary file 2 [file pone.0358431.s003.docx]

| **Variable** | **Patients** | **Comments** |
| --- | --- | --- |
| Discharge Disposition | 27 (0.25%) | Not in the primary model |
| Admitting Hospital | 16 (0.15%) | All of this information was subsequently recovered from the hospital-level admission record |
| Elixhauser Comorbidities | 1 (0.01%) | There were no codes recorded for this patient |
