# Supplementary material for: Persistent critical illness in acute hypoxic respiratory failure: A retrospective cohort study
Source: PLoS One. 2026 Sep 16;21(9):e0358431. doi: 10.1371/journal.pone.0358431 (PMC13580957; doi:10.1371/journal.pone.0358431)
Supplement: Supplementary file 3 [file pone.0358431.s004.docx]

| **Variable** | **All Patients (N=10,626)** | **Early Death (N=1,241)** | **PCI (N=3,107)** | **No PCI (N=6,278)** | **P Value** |
| --- | --- | --- | --- | --- | --- |
| **Demographics** |  |  |  |  |  |
| Age, mean (SD) | 60.9 (15.9) | 66.6 (15.4) | 59.6 (15.3) | 60.4 (16.0) | <.0001 |
| Female, n (%) | 4,467 (42.0) | 581 (46.8) | 1,302 (41.9) | 2,584 (41.2) | 0.001 |
| Race, n (%) |  |  |  |  | 0.039 |
| Asian | 553 (5.2) | 61 (4.9) | 186 (6.0) | 306 (4.9) |  |
| Black or African American | 783 (7.4) | 84 (6.8) | 255 (8.2) | 444 (7.1) |  |
| Other/Unknown | 627 (5.9) | 84 (6.8) | 186 (6.0) | 357 (5.7) |  |
| Non-Hispanic White | 8,663 (81.5) | 1,012 (81.5) | 2,480 (79.8) | 5,171 (82.4) |  |
| Tobacco Use, n (%) |  |  |  |  | <.0001 |
| Never | 3,449 (32.5) | 376 (30.3) | 971 (31.3) | 2,102 (33.5) |  |
| Quit | 3,615 (34.0) | 438 (35.3) | 1,007 (32.4) | 2,170 (34.6) |  |
| Unknown | 1,852 (17.4) | 238 (19.2) | 610 (19.6) | 1,004 (16.0) |  |
| Yes | 1,710 (16.1) | 189 (15.2) | 519 (16.7) | 1,002 (16.0) |  |
| **Elixhauser Comorbidities, n (%)** |  |  |  |  |  |
| Elixhauser Score, median (IQR) | 7 (0–16) | 10 (0–20) | 8 (0–16) | 6 (0–14) | <.0001 |
| HIV/AIDS† | 37 (0.3) | 1 (0.1) | 16 (0.5) | 20 (0.3) | — |
| Congestive Heart Failure | 2,058 (19.4) | 243 (19.6) | 657 (21.1) | 1,158 (18.4) | 0.008 |
| Valvular Disease | 1,956 (18.4) | 201 (16.2) | 499 (16.1) | 1,256 (20.0) | <.0001 |
| Pulmonary Circ Disorder | 1,150 (10.8) | 150 (12.1) | 356 (11.5) | 644 (10.3) | 0.067 |
| Peripheral Artery Disease | 1,885 (17.7) | 239 (19.3) | 528 (17.0) | 1,118 (17.8) | 0.205 |
| Hypertension | 6,208 (58.4) | 738 (59.5) | 1,797 (57.8) | 3,673 (58.5) | 0.601 |
| Paralysis | 393 (3.7) | 43 (3.5) | 131 (4.2) | 219 (3.5) | 0.192 |
| Neurological Disorder | 2,680 (25.2) | 323 (26.0) | 844 (27.2) | 1,513 (24.1) | 0.005 |
| Chronic Pulmonary Disease | 3,073 (28.9) | 342 (27.6) | 929 (29.9) | 1,802 (28.7) | 0.258 |
| Diabetes W/O Chronic Comp | 1,218 (11.5) | 127 (10.2) | 364 (11.7) | 727 (11.6) | 0.345 |
| Diabetes W/ Chronic Comp | 1,737 (16.3) | 200 (16.1) | 551 (17.7) | 986 (15.7) | 0.043 |
| Hypothyroidism | 1,292 (12.2) | 167 (13.5) | 374 (12.0) | 751 (12.0) | 0.329 |
| Renal Failure | 1,953 (18.4) | 313 (25.2) | 592 (19.1) | 1,048 (16.7) | <.0001 |
| Liver Disease | 1,526 (14.4) | 234 (18.9) | 460 (14.8) | 832 (13.3) | <.0001 |
| Peptic Ulcer Disease | 440 (4.1) | 62 (5.0) | 137 (4.4) | 241 (3.8) | 0.117 |
| Lymphoma | 246 (2.3) | 59 (4.8) | 76 (2.4) | 111 (1.8) | <.0001 |
| Metastatic Cancer | 841 (7.9) | 155 (12.5) | 200 (6.4) | 486 (7.7) | <.0001 |
| Solid Tumor W/O Metastasis | 1,124 (10.6) | 117 (9.4) | 277 (8.9) | 730 (11.6) | 0.0001 |
| Rheumatologic Disease | 755 (7.1) | 95 (7.7) | 224 (7.2) | 436 (6.9) | 0.650 |
| Coagulation Deficiency | 1,403 (13.2) | 231 (18.6) | 472 (15.2) | 700 (11.2) | <.0001 |
| Obesity | 2,295 (21.6) | 250 (20.1) | 709 (22.8) | 1,336 (21.3) | 0.098 |
| Weight Loss | 1,389 (13.1) | 188 (15.1) | 518 (16.7) | 683 (10.9) | <.0001 |
| Fluid & Electrolyte Disorder | 3,745 (35.2) | 541 (43.6) | 1,211 (39.0) | 1,993 (31.8) | <.0001 |
| Blood Loss Anemia | 304 (2.9) | 48 (3.9) | 92 (3.0) | 164 (2.6) | 0.049 |
| Deficiency Anemia | 2,966 (27.9) | 432 (34.8) | 940 (30.3) | 1,594 (25.4) | <.0001 |
| Alcohol Abuse | 1,466 (13.8) | 174 (14.0) | 451 (14.5) | 841 (13.4) | 0.326 |
| Drug Abuse | 1,081 (10.2) | 112 (9.0) | 348 (11.2) | 621 (9.9) | 0.052 |
| Psychoses | 1,188 (11.2) | 120 (9.7) | 367 (11.8) | 701 (11.2) | 0.129 |
| Depression | 3,107 (29.2) | 341 (27.5) | 931 (30.0) | 1,835 (29.2) | 0.266 |
| Cardiac Arrhythmia | 1,667 (15.7) | 181 (14.6) | 498 (16.0) | 988 (15.7) | 0.490 |
| **Admission Dx Category, n (%)** |  |  |  |  |  |
| Cardiovascular | 2,289 (21.5) | 343 (27.6) | 694 (22.3) | 1,252 (19.9) |  |
| Endocrine/Metabolic/Immunity | 383 (3.6) | 44 (3.5) | 103 (3.3) | 236 (3.8) |  |
| Factors Influencing Health | 1,551 (14.6) | 41 (3.3) | 296 (9.5) | 1,214 (19.3) |  |
| GI | 707 (6.7) | 98 (7.9) | 210 (6.8) | 399 (6.4) |  |
| Infectious Disease | 678 (6.4) | 193 (15.6) | 250 (8.0) | 235 (3.7) |  |
| Injury/Poisoning | 504 (4.7) | 53 (4.3) | 87 (2.8) | 364 (5.8) |  |
| Neurology | 529 (5.0) | 44 (3.5) | 149 (4.8) | 336 (5.4) |  |
| Oncology | 466 (4.4) | 50 (4.0) | 123 (4.0) | 293 (4.7) |  |
| Other | 1,117 (10.5) | 130 (10.5) | 371 (11.9) | 616 (9.8) |  |
| Pulmonary | 1,411 (13.3) | 156 (12.6) | 514 (16.5) | 741 (11.8) |  |
| Signs/Symptoms/Abn Findings | 991 (9.3) | 89 (7.2) | 310 (10.0) | 592 (9.4) |  |
| **Specific Admission Diagnoses, n (%)** |  |  |  |  |  |
| Cardiomyopathy/Pericarditis | 1,685 (15.9) | 190 (15.3) | 542 (17.4) | 953 (15.2) | 0.016 |
| Pulmonary Embolism | 346 (3.3) | 46 (3.7) | 153 (4.9) | 147 (2.3) | <.0001 |
| Pulmonary Vascular Disease | 1,044 (9.8) | 109 (8.8) | 331 (10.7) | 604 (9.6) | 0.121 |
| Cardiac Arrest | 1,206 (11.3) | 411 (33.1) | 379 (12.2) | 416 (6.6) | <.0001 |
| Heart Failure | 3,166 (29.8) | 385 (31.0) | 1,077 (34.7) | 1,704 (27.1) | <.0001 |
| Post-Proc Resp/Circ Failure | 920 (8.7) | 36 (2.9) | 458 (14.7) | 426 (6.8) | <.0001 |
| COVID-19 | 533 (5.0) | 73 (5.9) | 331 (10.7) | 129 (2.1) | <.0001 |
| Respiratory Failure | 7,483 (70.4) | 1,017 (82.0) | 2,529 (81.4) | 3,937 (62.7) | <.0001 |
| ARDS | 515 (4.8) | 67 (5.4) | 354 (11.4) | 94 (1.5) | <.0001 |
| Pneumonia | 3,666 (34.5) | 410 (33.0) | 1,758 (56.6) | 1,498 (23.9) | <.0001 |
| COPD | 1,895 (17.8) | 232 (18.7) | 570 (18.3) | 1,093 (17.4) | 0.377 |
| Asthma | 908 (8.5) | 60 (4.8) | 218 (7.0) | 630 (10.0) | <.0001 |
| Aspiration Pneumonitis | 1,618 (15.2) | 222 (17.9) | 623 (20.1) | 773 (12.3) | <.0001 |
| Other Lung Disease | 1,324 (12.5) | 114 (9.2) | 476 (15.3) | 734 (11.7) | <.0001 |
| Sepsis/Shock, n (%) |  |  |  |  | <.0001 |
| No Sepsis/Shock | 5,876 (55.3) | 420 (33.8) | 1,206 (38.8) | 4,250 (67.7) |  |
| Sepsis Only | 252 (2.4) | 23 (1.9) | 43 (1.4) | 186 (3.0) |  |
| Septic Shock | 4,498 (42.3) | 798 (64.3) | 1,858 (59.8) | 1,842 (29.3) |  |
| Lung Transplant Complication | 308 (2.9) | 4 (0.3) | 101 (3.3) | 203 (3.2) |  |
| **Clinical Variables** |  |  |  |  |  |
| ICU Source: Ward, n (%) | 4,341 (40.9) | 480 (38.7) | 1,359 (43.7) | 2,502 (39.9) | 0.0004 |
| Primary Service: Surgery, n (%) | 5,828 (54.8) | 398 (32.1) | 1,680 (54.1) | 3,750 (59.7) | <.0001 |
| LAPS2, mean (SD) | 156.3 (52.0) | 203.5 (52.9) | 160.2 (52.6) | 145.0 (45.7) | <.0001 |
| Pre-ICU LOS, median days (IQR) | 0 (0–1) | 0 (0–2) | 0 (0–2) | 0 (0–1) | 0.001 |
| Code Status, n (%) |  |  |  |  | <.0001 |
| DNI or DNR | 433 (4.1) | 155 (12.5) | 183 (5.9) | 95 (1.5) |  |
| DNI/DNR | 1,419 (13.4) | 647 (52.1) | 524 (16.9) | 248 (4.0) |  |
| Full Code | 8,774 (82.6) | 439 (35.4) | 2,400 (77.2) | 5,935 (94.5) |  |
| **Outcomes** |  |  |  |  |  |
| MV Duration, median days (IQR) | 2 (1–7) | 3 (1–5) | 10 (6–18) | 1 (1–3) | <.0001 |
| Hospital LOS, median days (IQR) | 11 (6–19) | 5 (2–8) | 22 (16–31) | 9 (6–14) | <.0001 |
| Discharge Disposition, n (%) |  |  |  |  | <.0001 |
| Home | 4,186 (39.4) | 0 (0) | 649 (20.9) | 3,537 (56.3) |  |
| SNF | 2,147 (20.2) | 5 (0.4) | 577 (18.6) | 1,565 (24.9) |  |
| Expired/Hospice | 2,263 (21.3) | 1,229 (99.0) | 821 (26.4) | 213 (3.4) |  |
| LTACH | 752 (7.1) | 0 (0) | 618 (19.9) | 134 (2.1) |  |
| Acute Inpatient Rehab | 777 (7.3) | 0 (0) | 351 (11.3) | 426 (6.8) |  |
| Another Hospital | 220 (2.1) | 7 (0.6) | 47 (1.5) | 166 (2.6) |  |
| Other/Unknown | 281 (2.6) | 0 (0) | 44 (1.4) | 237 (3.8) |  |
| In-Hospital Mortality, n (%) | 2,147 (20.2) | 1,224 (98.6) | 773 (24.9) | 150 (2.4) | <.0001 |
| One-Year Mortality, n (%) | 3,086 (29.0) | 1,241 (100.0) | 1,113 (35.8) | 732 (11.7) | <.0001 |
