# Supplementary material for: Persistent critical illness in acute hypoxic respiratory failure: A retrospective cohort study
Source: PLoS One. 2026 Sep 16;21(9):e0358431. doi: 10.1371/journal.pone.0358431 (PMC13580957; doi:10.1371/journal.pone.0358431)
Supplement: Supplementary file 4 [file pone.0358431.s005.docx]

| **Parameter** | **Level** | **Model 2A OR (95% CI)** | **P** | **Model 2B OR (95% CI)** | **P** |
| --- | --- | --- | --- | --- | --- |
| Gender | Female vs Male | 0.84 (0.71–1.00) | 0.049 | 1.09 (0.98–1.21) | 0.119 |
| Age (per year) |  | 0.96 (0.96–0.97) | <.0001 | 1.00 (0.99–1.00) | 0.039 |
| ***Race (ref: White)*** |  |  |  |  |  |
|  | Asian | 1.37 (0.94–2.01) | 0.102 | 0.95 (0.75–1.19) | 0.632 |
|  | Black | 1.14 (0.82–1.59) | 0.433 | 1.03 (0.85–1.25) | 0.767 |
|  | Other/Unknown | 0.80 (0.57–1.13) | 0.209 | 0.90 (0.73–1.12) | 0.336 |
| ***Tobacco Use (ref: Never)*** |  |  |  |  |  |
|  | Quit | 0.95 (0.77–1.18) | 0.647 | 1.01 (0.89–1.15) | 0.875 |
|  | Unknown | 1.21 (0.94–1.56) | 0.137 | 1.12 (0.95–1.31) | 0.171 |
|  | Yes | 0.95 (0.72–1.26) | 0.739 | 1.12 (0.95–1.32) | 0.178 |
| ***Elixhauser Comorbidities*** |  |  |  |  |  |
| HIV/AIDS |  | 3.18 (0.38–26.70) | 0.286 | 1.71 (0.82–3.55) | 0.152 |
| CHF |  | 0.95 (0.73–1.25) | 0.729 | 1.07 (0.91–1.26) | 0.417 |
| Valvular Disease |  | 0.94 (0.73–1.22) | 0.651 | 0.84 (0.72–0.97) | 0.021 |
| Pulmonary Circ Disorder |  | 0.89 (0.66–1.19) | 0.427 | 0.96 (0.80–1.16) | 0.695 |
| PAD |  | 0.93 (0.73–1.17) | 0.516 | 0.99 (0.86–1.14) | 0.866 |
| Hypertension |  | 1.07 (0.87–1.31) | 0.509 | 0.95 (0.84–1.07) | 0.413 |
| Paralysis |  | 0.90 (0.57–1.41) | 0.634 | 0.91 (0.70–1.18) | 0.479 |
| Neurological Disorder |  | 1.07 (0.87–1.31) | 0.520 | 1.08 (0.95–1.22) | 0.234 |
| Chronic Pulm Disease |  | 1.02 (0.80–1.28) | 0.900 | 1.11 (0.96–1.28) | 0.145 |
| Diabetes W/O Chronic Comp |  | 1.25 (0.95–1.65) | 0.105 | 1.02 (0.87–1.19) | 0.858 |
| Diabetes W/ Chronic Comp |  | 1.38 (1.08–1.78) | 0.011 | 0.99 (0.85–1.15) | 0.906 |
| Hypothyroidism |  | 0.96 (0.75–1.25) | 0.776 | 0.93 (0.80–1.09) | 0.391 |
| Renal Failure |  | 0.79 (0.62–0.99) | 0.041 | 0.97 (0.84–1.13) | 0.707 |
| Liver Disease |  | 0.70 (0.55–0.90) | 0.005 | 0.94 (0.81–1.11) | 0.470 |
| Peptic Ulcer Disease |  | 0.96 (0.64–1.42) | 0.827 | 1.02 (0.80–1.31) | 0.876 |
| Lymphoma |  | 0.57 (0.37–0.88) | 0.011 | 1.12 (0.79–1.58) | 0.533 |
| Metastatic Cancer |  | 0.52 (0.39–0.69) | <.0001 | 0.71 (0.58–0.87) | <.001 |
| Solid Tumor W/O Mets |  | 1.05 (0.79–1.41) | 0.724 | 0.76 (0.63–0.90) | 0.002 |
| Rheumatologic Disease |  | 0.90 (0.65–1.24) | 0.514 | 0.88 (0.72–1.07) | 0.211 |
| Coagulation Deficiency |  | 0.94 (0.74–1.20) | 0.614 | 1.17 (1.00–1.38) | 0.054 |
| Obesity |  | 1.11 (0.89–1.39) | 0.337 | 1.02 (0.90–1.17) | 0.724 |
| Weight Loss |  | 1.37 (1.07–1.76) | 0.013 | 1.27 (1.09–1.48) | 0.003 |
| Fluid & Electrolyte Disorder |  | 0.99 (0.82–1.21) | 0.940 | 1.08 (0.96–1.22) | 0.197 |
| Blood Loss Anemia |  | 1.01 (0.64–1.60) | 0.959 | 0.86 (0.63–1.16) | 0.315 |
| Deficiency Anemia |  | 1.04 (0.83–1.29) | 0.761 | 1.09 (0.96–1.25) | 0.185 |
| Alcohol Abuse |  | 1.19 (0.91–1.56) | 0.203 | 1.10 (0.93–1.29) | 0.259 |
| Drug Abuse |  | 1.08 (0.79–1.48) | 0.617 | 0.99 (0.83–1.19) | 0.950 |
| Psychoses |  | 1.19 (0.89–1.58) | 0.246 | 1.02 (0.86–1.21) | 0.819 |
| Depression |  | 1.05 (0.85–1.30) | 0.647 | 0.93 (0.82–1.05) | 0.237 |
| Cardiac Arrhythmia |  | 1.32 (1.02–1.71) | 0.037 | 1.16 (1.00–1.36) | 0.055 |
| ***Admission Dx Category (ref: CV)*** |  |  |  |  |  |
|  | Endocrine/Metabolic | 1.03 (0.64–1.66) | 0.894 | 0.71 (0.53–0.95) | 0.021 |
|  | Factors Infl Health | 2.15 (1.42–3.25) | 0.0003 | 0.45 (0.38–0.54) | <.0001 |
|  | GI | 1.01 (0.71–1.45) | 0.946 | 0.87 (0.69–1.09) | 0.211 |
|  | ID | 0.76 (0.56–1.04) | 0.088 | 1.10 (0.86–1.39) | 0.452 |
|  | Injury/Poisoning | 0.73 (0.45–1.17) | 0.192 | 0.54 (0.41–0.72) | <.0001 |
|  | Neurology | 1.67 (1.06–2.63) | 0.028 | 0.98 (0.77–1.25) | 0.876 |
|  | Oncology | 0.88 (0.54–1.43) | 0.605 | 0.83 (0.62–1.11) | 0.203 |
|  | Other | 1.15 (0.84–1.57) | 0.382 | 0.89 (0.74–1.08) | 0.236 |
|  | Pulmonary | 1.22 (0.90–1.64) | 0.197 | 0.76 (0.63–0.92) | 0.004 |
|  | Signs/Sx/Abn Findings | 1.68 (1.21–2.35) | 0.002 | 0.84 (0.69–1.02) | 0.084 |
| ***Specific Diagnoses*** |  |  |  |  |  |
| Cardiomyopathy/Pericarditis |  | 1.06 (0.83–1.34) | 0.648 | 1.04 (0.89–1.20) | 0.641 |
| Pulmonary Embolism |  | 1.29 (0.84–2.00) | 0.245 | 1.34 (1.01–1.78) | 0.044 |
| Pulmonary Vascular Disease |  | 1.10 (0.81–1.49) | 0.560 | 1.15 (0.96–1.37) | 0.125 |
| Cardiac Arrest |  | 0.26 (0.21–0.32) | <.0001 | 1.33 (1.11–1.59) | 0.002 |
| Heart Failure |  | 1.43 (1.16–1.78) | 0.001 | 1.23 (1.07–1.41) | 0.004 |
| Post-Proc Resp/Circ Failure |  | 3.69 (2.49–5.46) | <.0001 | 1.53 (1.29–1.82) | <.0001 |
| COVID-19 |  | 1.24 (0.87–1.76) | 0.246 | 2.65 (2.04–3.43) | <.0001 |
| Respiratory Failure |  | 1.09 (0.85–1.39) | 0.507 | 1.97 (1.71–2.26) | <.0001 |
| ARDS |  | 1.58 (1.09–2.28) | 0.015 | 4.24 (3.23–5.57) | <.0001 |
| Pneumonia |  | 2.35 (1.94–2.84) | <.0001 | 2.78 (2.49–3.11) | <.0001 |
| COPD |  | 1.04 (0.81–1.34) | 0.759 | 0.93 (0.80–1.09) | 0.362 |
| Asthma |  | 1.22 (0.83–1.78) | 0.316 | 0.70 (0.58–0.86) | <.001 |
| Aspiration Pneumonitis |  | 1.64 (1.32–2.04) | <.0001 | 1.49 (1.30–1.70) | <.0001 |
| Other Lung Disease |  | 1.16 (0.89–1.53) | 0.276 | 1.35 (1.15–1.58) | 0.0002 |
| Sepsis/Shock (ref: No Sepsis) |  |  |  |  |  |
| Sepsis Only |  | 1.10 (0.57–2.11) | 0.779 | 1.98 (1.37–2.86) | 0.0003 |
| Septic Shock |  | 1.28 (1.05–1.56) | 0.014 | 2.09 (1.87–2.34) | <.0001 |
| Lung Transplant Complication |  | 3.26 (1.10–9.65) | 0.033 | 0.91 (0.66–1.26) | 0.584 |
| ***Other Covariates*** |  |  |  |  |  |
| ICU Source | Ward vs ED | 0.98 (0.80–1.19) | 0.826 | 1.00 (0.89–1.13) | 0.984 |
| Primary Service | Medicine vs Surgery | 0.29 (0.24–0.36) | <.0001 | 0.57 (0.50–0.65) | <.0001 |
| LAPS2 (per point) |  | 0.988 (0.986–0.989) | <.0001 | 1.002 (1.001–1.003) | 0.004 |
| Pre-ICU LOS (per day) |  | 0.96 (0.95–0.98) | <.0001 | 1.03 (1.02–1.05) | <.0001 |
