# Supplementary material for: Persistent critical illness in acute hypoxic respiratory failure: A retrospective cohort study
Source: PLoS One. 2026 Sep 16;21(9):e0358431. doi: 10.1371/journal.pone.0358431 (PMC13580957; doi:10.1371/journal.pone.0358431)
Supplement: Supplementary file 6 [file pone.0358431.s007.docx]

| **Parameter** | **Level** | **HR (95% CI)** | **P Value** |
| --- | --- | --- | --- |
| Age (per year) |  | 1.03 (1.03–1.03) | <.0001 |
| Race (ref: White) |  |  |  |
|  | Asian | 1.00 (0.85–1.19) | 0.9360 |
|  | Black | 0.94 (0.81–1.08) | 0.3723 |
|  | Other/Unknown | 1.22 (1.05–1.42) | 0.0090 |
| ICU Source | Ward vs ED | 1.15 (1.06–1.26) | 0.001 |
| Primary Service | Medicine vs Surgery | 1.98 (1.75–2.08) | <.0001 |
| PerCI 7 day Cutoff | Yes vs No | 2.67 (2.40–2.96) | <.0001 |
