# Supplementary material for: Persistent critical illness in acute hypoxic respiratory failure: A retrospective cohort study
Source: PLoS One. 2026 Sep 16;21(9):e0358431. doi: 10.1371/journal.pone.0358431 (PMC13580957; doi:10.1371/journal.pone.0358431)
Supplement: Supplementary file 7 [file pone.0358431.s008.docx]

| **Variable** | **All Patients (N=10,626)** | **Early Death (N=3,086)** | **Alive 1yr After ICU Start**  **(N=7,540)** | **P Value** |
| --- | --- | --- | --- | --- |
| Age, mean (SD) | 60.9 (15.9) | 65.8 (15.0) | 58.8 (15.8) | <.0001 |
| Race, n (%) |  |  |  | 0.5801 |
| Asian | 553 (5.2) | 170 (5.5) | 383 (5.1) |  |
| Black or African American | 783 (7.4) | 216 (7.0) | 567 (7.5) |  |
| Other/Unknown | 627 (5.9) | 189 (6.1) | 438 (5.8) |  |
| Non-Hispanic White | 8,663 (81.5) | 2511 (81.4) | 6152 (81.6) |  |
| ICU Source: Ward, n (%) | 4,341 (40.9) | 1371 (44.4) | 2970 (39.4) | <.0001 |
| Primary Service: Surgery, n (%) | 5,828 (54.8) | 1245 (40.3) | 4583 (60.8) | <.0001 |
